# Associations of Cone Density and Choroidal Thickness with Age: A Cross-Sectional Study

**DOI:** 10.1016/j.xops.2026.101329

**Published:** 2026-07-23

**Authors:** Jue Lin, Aixia Yao, Tong Wang, Ning Xu, Shenao Yi, Yang Gao, Ruyu Yan, Jie Ye, Yiyi Wang, Meixiao Shen, Chenxiao Wang, Fan Lu, Yilei Shao

**Affiliations:** 1 Zhejiang Key Laboratory of Ophthalmic Drug Discovery and Medical Device Research, Eye Hospital, Wenzhou Medical University, Wenzhou, China; 2 National Engineering Research Center of Ophthalmology and Optometry, Eye Hospital, Wenzhou Medical University, Wenzhou, China; 3 National Clinical Research Center for Ocular Diseases, Eye Hospital, Wenzhou Medical University, Wenzhou, China; 4 The Affiliated Eye Hospital, Nanjing Medical University, Nanjing, China

**Keywords:** Cone density, Choroidal thickness, Visual acuity, Age

## Abstract

**Purpose:**

To investigate the interrelationships among choroidal blood supply, photoreceptor structure, and visual function during normal aging using OCT and adaptive optics (AO).

**Design:**

A cross-sectional study.

**Subjects:**

A total of 68 participants aged 20–70 years were included in this study.

**Methods:**

All enrolled participants underwent a complete ophthalmological examination, including best-corrected visual acuity, axial length, OCT, and AO fundus photography. Photoreceptor images captured by AO imaging were used to calculate the cone density. A choroidal thickness map was obtained from OCT images using a custom algorithm.

**Main Outcome Measures:**

Detailed analysis was performed for the differences and correlations among cone density, choroidal thickness, and age.

**Results:**

Compared with group 1 (G1, aged 20–34 years), the cone densities of group 3 (G3, aged 50–64 years) and group 4 (G4, aged 65–79 years) were significantly lower at 0.5 mm to 1.1 mm eccentricities (all *P* < 0.05). The mean ± standard deviation cone densities (×1000 cells/mm^2^) at 0.5, 0.7, 0.9, and 1.1 mm eccentricities were 27.86 ± 2.77, 22.43 ± 2.04, 18.91 ± 1.42, 16.95 ± 1.19 for G1, 25.33 ± 2.44, 21.52 ± 2.02, 18.86 ± 1.89, 17.05 ± 1.29 for G3, and 22.29 ± 2.10, 20.30 ± 2.05, 17.50 ± 1.67, 15.56 ± 1.45 for G4, respectively. The choroidal thicknesses of group2 (G2, aged 35∼49 years), G3, and G4 were significantly thinner (G2: 199.45 ± 15.87 μm, G3: 156.87 ± 13.45 μm,G4: 142.25 ± 20.98 μm, all *P* < 0.05) than G1 (222.20 ± 40.41 μm). The cone density at all eccentricities was significantly correlated with choroidal thickness (all *P* < 0.05).

**Conclusions:**

The cones showed significant correlation with the choroid and age. Reduced choroidal thickness and older age were associated with lower cone density, highlighting the interdependent aging of vascular and structural parameters in age-related visual impairment.

**Financial Disclosure(s):**

The author has no/the authors have no proprietary or commercial interest in any materials discussed in this article.

The integrity of macular cone photoreceptor structures and their functions are crucial for the formation and maintenance of human vision.^1^^,^^2^ Both histological studies and in vivo adaptive optics (AO) investigations have reported a decline in cone photoreceptors with advancing age.^3^^,^^4^ Photoreceptor degeneration is considered a key component of the mechanisms driving age-related chorioretinopathies, such as age-related macular degeneration and diabetic retinopathy, and eventually causing irreversible visual impairment and blindness in the elderly.^5^, ^6^, ^7^

Diminished choroidal perfusion may contribute to damage in cone photoreceptor cells.^8^ The choroid constitutes a vital vascular structure within the eye, responsible for delivering nutrients and eliminating waste products from photoreceptor cells.^8^ Research utilizing structural OCT and OCT angiography has revealed a reduction in choroidal thickness and choriocapillaris over time, potentially leading to cone injury.^9^, ^10^, ^11^, ^12^ Consequently, investigating age-related alterations in photoreceptors and choroids will offer valuable insights into the physiological and pathophysiological mechanisms underlying age-related chorioretinopathy.

Previously, we employed AO and OCT methodologies to examine the relationship between visual impairment and structural modifications in the retina and choroid in patients with high myopia.^13^^,^^14^ The present study aims to elucidate the characteristics of age-related changes in cone density and choroidal thickness and to analyze the associations of these alterations by using OCT and AO techniques.

## Methods

### Participants

This cross-sectional study enrolled healthy participants aged ≥20 years at the Eye Hospital of Wenzhou Medical University in November 2018. The Ethics Committee of Wenzhou Medical University (trial registration code: 2021-014-K-11) approved the project, and all participants provided written informed consent after being apprised of the benefits, risks, and potential adverse outcomes of the procedures. The study adhered to the principles outlined in the Declaration of Helsinki.

A comprehensive ophthalmological examination was conducted for all participants (between 2:00 and 4:00 pm), which included subjective refraction, best-corrected visual acuity (BCVA) using the logarithmic visual acuity chart, intraocular pressure assessment with the Full Auto Tonometer TX-F (Topcon), axial length (AL, defined as the distance from the anterior corneal surface to the photoreceptor outer segment layer at the fovea, representing the total anatomical length of the eye.) measurement using IOL Master biometry (IOL Master; Carl Zeiss Meditec), slit-lamp examination, and fundus photography (Canon EOS 10 SLR backing; Canon, Inc). Only the right eye of each subject was included and subjected to further examinations. Exclusion criteria comprised a history of ocular surgery, relevant ocular diseases (e.g., age-related macular degeneration, pathological myopia, diabetic retinopathy), severe cataracts, other optic media opacities, diabetes mellitus, uncontrolled hypertension, or additional cardiovascular diseases. Participants with greater than +3.00 or –3.00 diopters of a spherical equivalent were excluded to eliminate the effect of axial elongation.

Based on age, participants were divided into 4 groups: group 1 (G1) included individuals aged 20–34 years, group 2 (G2) comprised those aged 35–49 years, group 3 (G3) encompassed participants aged 50–64 years, and group 4 (G4) consisted of participants aged 65-79 years old.

### Adaptive Optics Fundus Camera Imaging and Analysis

**Figure 1:**
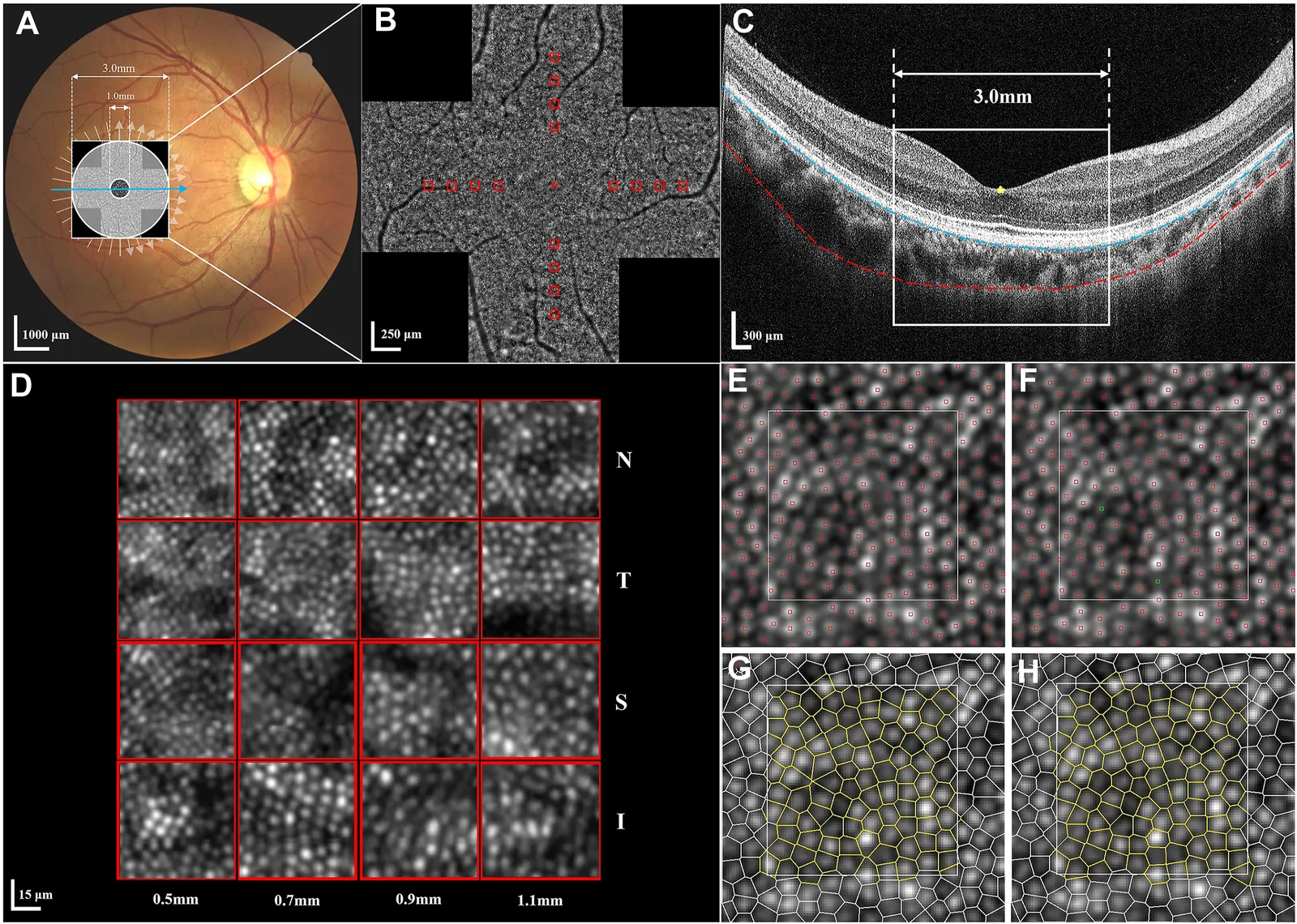
Imaging with AO Fundus Camera and OCT. **(A)** Adaptive optics montage superimposed on the fundus image. Eighteen consecutive B-scans of transverse section images of the fundus were captured using an 8-mm radial line scanning centered on the fovea. The concentric ring ranging from 0.3 mm to 1.5 mm from the fovea represents the analysis area. **(B)** The red asterisk centers the AO montage with a 10° × 10° window size. Regions of interest (ROIs), 80 × 80 pixels or 0.2° × 0.2°, were selected for analysis at eccentricities of 0.6 mm and 1.2 mm from the fovea: 1.67° (0.5 mm), 2.34° (0.7 mm), 3.01° (0.9 mm), and 3.68° (1.1 mm) in the right eye. **(C)** The OCT image along the horizontal meridian, represented by the blue arrow in **(A)**. The bar measures 15 μm. The axial lengths (ALs) of the eyes were 22.90 mm. **(D)** Magnified view of ROIs on the nasal meridian. **(E and G)** Automatic detection of the cones (the 0.5 mm eccentricity, nasal region). **(F and H)** The cone detection after manual correction. The green dots represent cones that have been manually corrected. AO = adaptive optics; I = inferior; N = nasal; S = superior; T = temporal. (Image from: a female, 26 years old, AL = 22.90 mm).

High-resolution photoreceptor images were captured using an AO fundus camera (AOimage 3.1, rtx1, Imagine Eyes), which primarily comprised a Shack–Hartmann wavefront sensor, deformable mirror, and high-resolution fundus camera. The digital resolution of the raw image is that each pixel corresponds to 1.6 μm of actual retinal distance. The lateral optical resolution was 2.0 μm, with an approximate field of view of 4.2° × 4.2°. Prior to AO imaging, each participant's right eye was dilated through the combined use of a single drop of 0.5% tropicamide and 10% phenylephrine hydrochloride. An additional drop of tropicamide may be administered with a 10-minute interval following evaluation when the pupil diameter remained small. Adaptive optics imaging would be performed when the pupil diameter stabilizes at ≥6 mm. Participants were instructed to maintain stable fixation by gazing at the built-in yellow cross target and then shifting to the pre-set coordinates. First, participants focused on the central region (0, 0°). A video comprising 40 frames was recorded, and a built-in algorithm was employed to correlate and average the captured frames, yielding a final image with a 4° × 4° field of view. Additional perifoveal areas were imaged by guiding participants to sequentially fixate at 3° of eccentricity along the 4 meridians (superior [S], nasal [N], temporal [T], and inferior [I]). Ultimately, 5 images of adjacent regions from each subject were merged using i2k Retina software (version 2.6.0) to create a montage (cross shape with an approximate 10° × 10° field, Fig 1A).

A single operator (A.X.) calculated cone density using image processing and recognition software provided by the manufacturer (AO Detect 2.0b13; Imaging Eyes). The operator was blinded to participant age groups, as all images were randomly recoded before analysis. Axial length correction was applied to adjust retinal imaging magnification based on Bennett's formula, t = p × q × s, where t represents the actual scan length, p is the magnification factor of the camera in the OCT and AO imaging system, and s denotes the original image measurement.^13^ Regions of interest (ROIs) consisting of 80 × 80 pixels, corresponding to a 62 × 62 μm region on the retina of emmetropic eyes, were chosen for cone density analysis at 0.5 mm, 0.7 mm, 0.9 mm, and 1.1 mm from the fovea along the temporal, nasal, superior, and inferior meridians of the right eye following magnification correction (Fig 1B). During the image detection process, we also performed manual correction of the cone recognition (Fig 1E–H), and very few (5 eyes) needed to be corrected manually. Two researchers (J.L. and W.T.) were responsible for verifying the results recognized by the software to reduce errors caused by the recognition software. While a formal intergrader reliability test was not conducted in this specific cohort, the cone counting method utilized relies on a largely automated algorithm with minimal operator intervention, with its reproducibility having been previously established by the manufacturer.^15^^,^^16^ Visual field angle to actual retinal distance conversion outcomes are in accordance with the simplified standard (1° ≈ 288 to 300 μm), which ensures accurate correlation between the position in the visual field and the corresponding physical retinal region.

### Measurement of Choroid Thicknesses with OCT

The chorioretinal profile was imaged using spectral-domain OCT (Optovue RTVue XR Avanti; Optovue Inc). Eighteen consecutive B-scans of the transverse section images of the fundus were captured using an 8-mm radial line scan centered on the fovea, generating 3-dimensional maps of chorioretinal thickness. Bennett's formula,^17^ based on AL, was employed to correct image magnification before calculating choroidal thickness, which was defined as the vertical distance from the lower border of the hyperreflective retinal pigment epithelium to the choroid-scleral junction. Choroidal thickness was automatically detected using custom-developed software.^13^ For analysis, the choroidal thickness map was presented as a 3.0-mm-diameter region centered on the fovea (Fig 1C). The thickness of the choroid in this region was calculated after excluding the central zone (diameter = 0.6 mm), resulting in a 2.4-mm-diameter annular region used for further analysis. This region was then divided into 4 subfields: nasal, temporal, superior, and inferior (N, T, S, and I, respectively). The criteria for recognizing segmentation errors have been reported in our previous research.^18^^,^^19^ Manual corrections were required if errors occurred during automatic segmentation, such as the occasional misidentification of the posterior choroidal boundary in emmetropic eyes. Such manual intervention was necessary in approximately 5% of the eyes.

### Statistical Methods

Statistical analyses were performed using Statistical Software (version 23.0; SPSS Inc). One-way analysis of variance was performed to compare differences in age, logarithm of the minimum angle of resolution, spherical equivalent, and AL among the 4 groups. Sex differences were determined using a χ^2^ test. The choroidal thickness and cone density were analyzed after magnification correction of AO or OCT images. To evaluate age as a continuous variable, Pearson correlation analyses were conducted to assess the relationships among age, choroidal thickness, cone density, and BCVA. Pearson correlation assumptions (linearity, normality, no outliers, independence) were verified and met. Both cone density within each meridian at 0.5–1.1 mm eccentricities and choroidal thickness across meridians in the 4 age groups were analyzed using a generalized linear model, when the influences of AL and gender on the choroid and cone were included as covariates to control for and minimize the confounding effects. Statistical significance was set at *P* < 0.05.

## Results

### Participant Characteristics

We analyzed 68 healthy participants aged 20 to 70 years. Demographic information is listed in Table 1. Significant differences in age, spherical equivalent, logarithm of the minimum angle of resolution, and AL were observed among the 4 groups (*P* < 0.05).

**Table 1:** Baseline Data for 4 Groups of Different Age (n = 68)

	Group 1	Group 2	Group 3	Group 4	*P* Value
Eyes/participants	22	17	19	10	
Female/male	15/7	6/11	15/4	9/1	0.011†
Age (years, mean ± SD)	24.77 ± 2.78	41.84 ± 3.54	55.87 ± 2.96	67.63 ± 2.20	<0.001∗
(Range)	20 ∼ 33	36 ∼ 47	50 ∼ 60	65 ∼ 70	
logMAR (mean ± SD)	–0.07 ± 0.05	–0.08 ± 0.05	–0.01 ± 0.05	0.06 ± 0.04	<0.001∗
(range)	–0.18 ∼ 0.00	–0.18 ∼ 0.00	–0.08 ∼ 0.10	0.08 ∼ 0.10	
SE (D, mean ± SD)	–1.47 ± 1.20	–0.51 ± 0.76	–0.04 ± 0.27	–0.03 ± 0.36	<0.001∗
(Range)	–3.00 ∼ +0.50	–2.00 ∼ +0.25	–0.75 ∼ +0.50	–0.50 ∼ +0.63	
AL (mm, mean ± SD)	23.99 ± 0.89	23.30 ± 0.92	22.99 ± 0.70	23.07 ± 0.48	0.001∗
(range)	22.39 ∼ 25.35	20.99 ∼ 24.83	21.70 ∼ 24.52	22.33 ∼ 23.73	

AL = axial length; ANOVA = analysis of variance; D = diopters; logMAR = logarithm of the minimum angle of resolution; SD = standard deviation; SE = spherical equivalent.

∗ ANOVA.

† Standard chi square test.

### Differences in Cone Density at Each Eccentricity along 4 Meridians among the 4 Groups

**Figure 2:**
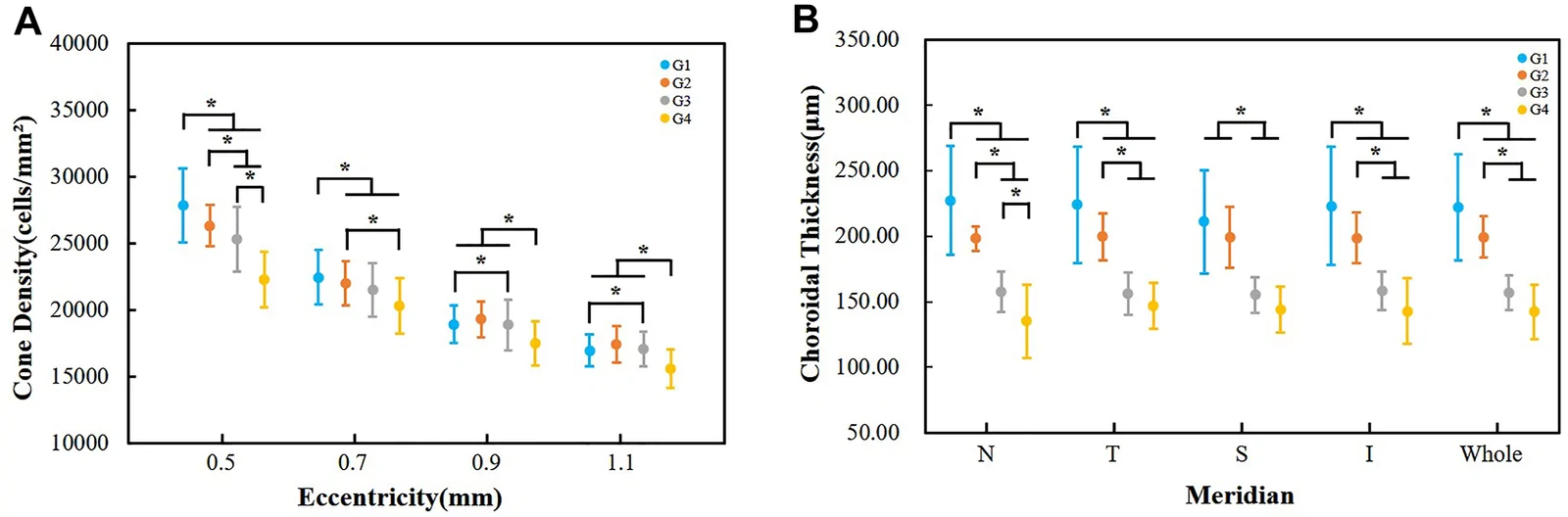
Choroid thickness and cone density for the different age groups. **(A)** Cone density for the different age groups in different eccentricities. Compared to G1, G2 had lower cone density only at 0.5 mm and 0.7 mm eccentricity. G3 and G4 had a significantly lower average cone density than G1 at all the eccentricities. G3 only showed lower cone density than G2 at 0.5 mm eccentricity. G4 showed significant difference with G2 and G3, except the 0.7 mm eccentricity of G3. **(B)** Choroid thickness for 4 age groups in different meridians. Compared to G1, G2, G3 and G4 manifested significant thinning of choroid. G4, G3 had a significant thinner of choroid than G2. Compared to G1, G2 manifested significant thinning except the superior meridian. G4 manifested significant thinning of nasal choroid compared with G3. ∗ represented *P* < 0.05. Bars = standard deviation. I = inferior; N = nasal; S = superior; T = temporal.

The cone density at each eccentricity was averaged from 4 meridians and compared among the different groups (Supplementary Table S1, available at www.ophthalmologyscience.org, and Fig 2A). Generally, cone density was highest at 0.5 mm eccentricity, with values of 27.86 ± 2.77 (20–34 years), 26.31 ± 1.54 (35–49 years), 25.33 ± 2.44 (50–64 years), and 22.29 ± 2.10 (65–79 years) ×1000 cells/mm^2^, and declined rapidly with increasing retinal eccentricity. At 0.7 mm, 0.9 mm, and 1.1 mm eccentricities, the mean cone densities were 22.43 ± 2.04, 18.91 ± 1.42, 16.95 ± 1.19 (20–34 years); 22.01 ± 1.66, 19.28 ± 1.37, 17.40 ± 1.38 (35–49 years); 21.52 ± 2.02, 18.86 ± 1.89, 17.05 ± 1.29 (50–64 years); and 20.30 ± 2.05, 17.50 ± 1.67, 15.56 ± 1.45 (65–79 years), all ×1000 cells/mm^2^, respectively. Briefly, after correcting for AL and sex using a generalized linear model analysis, individuals aged 35–49 years exhibited lower cone density than those aged 20–34 years at 0.5 mm and 0.7 mm eccentricity (0.5 mm: B = 2685.92, *P* < 0.001; 0.7 mm: B = 1788.23, *P* = 0.002, respectively). Those aged 50–64 years showed lower cone density than the 35–49 years group only at 0.5 mm eccentricity (B = 1352.50, *P* = 0.049). In comparison with the 20–34 years group, participants aged 50–64 years and 65–79 years had significantly lower cone densities at all eccentricities (all *P* < 0.05; detailed B values are presented in Supplementary Table S4A, available at www.ophthalmologyscience.org). Individuals aged 65–79 years demonstrated a significant difference compared to the 35–49 years group at all eccentricities (all *P* < 0.05, detailed B values are presented in Supplementary Table S4A) and showed lower cone densities than the 50–64 years group at all eccentricities except 0.7 mm (0.5 mm: B = 2891.46, *P* < 0.001; 0.9 mm: B = 1168.14, *P* = 0.02; 1.1 mm: B = 1324.28, *P* = 0.001).

Detailed differences in cone density among the 4 groups along the 4 meridians and 4 eccentricities are shown in Supplementary Figure S1 and Table S1, available at www.ophthalmologyscience.org. At 0.5 mm eccentricity, individuals aged 20–34 years generally exhibited higher cone density than the older groups. Compared to individuals aged 20–34 years, the 35–49 years group showed significantly lower cone density in the temporal (B = 2676.53, *P* = 0.002), superior (B = 2558.59, *P* < 0.001), and inferior meridians (B = 3536.93, *P* = 0.005), but not in the nasal meridian (B = 1906.78, *P* = 0.060). Both the 50–64 years group and the 65-79 years group had significantly lower cone density than those aged 20–34 years across all 4 meridians (all *P* < 0.001; detailed B values are presented in Supplementary Table S4A). Participants aged 35–49 years and 50–64 years showed differences in cone density only along the nasal meridian (B = 2732.45, *P* = 0.006). Furthermore, those aged 65–79 years had a significantly lower cone density than the 50–64 years group across all meridians except the inferior meridian (nasal: B = 2930.10, *P* = 0.008; temporal: B = 4029.15, *P* < 0.001; superior: B = 2401.34, *P* = 0.001). At 0.7 mm eccentricity, significant differences in cone density were found along the nasal meridian between the 20–34 years group and all 3 older groups (G2–G1: B = 2180.38, *P* = 0.023; G3–G1: B = 2949.04, *P* = 0.001; G4–G1: B = 4794.83, *P* < 0.001). Along the superior meridian, both the 50–64 years and 65-79 years groups had significantly lower cone density than the 20–34 years group (G3–G1: B = 1502.36, *P* = 0.025; G4–G1: B = 1939.23, *P* = 0.013). Along the temporal meridian, the 50–64 years and 65–79 years groups showed significantly lower cone density than the 20–34 years group (G3–G1: B = 1549.77, *P* = 0.041; G4–G1: B = 3676.39, *P* < 0.001), and the 65-79 years group also had lower density than the 35–49 years and 50–64 years groups (G4–G2: B = 2380.13, *P* = 0.008; G4–G3: B = 2126.62, *P* = 0.011). Along the inferior meridian, the 35–49 years and 50–64 years groups exhibited significantly lower cone density than the 20–34 years group (G2–G1: B = 2502.05, *P* = 0.021; G3–G1: B = 2283.89, *P* = 0.026). At 0.9 mm eccentricity, along the temporal meridian, the 35–49 years, 50–64 years, and 65-79 years groups exhibited significantly lower cone densities than the 20–34 years group (G2–G1: B = 1935.30, *P* = 0.007; G3–G1: B = 1858.92, *P* = 0.007; G4–G1: B = 3069.40, *P* < 0.001). At this eccentricity, only the 65-79 years group showed a significant difference in cone density from the other 3 groups along the nasal meridians (G4–G1: B = 3297.39, *P* < 0.001; G4–G2: B = 3265.22, *P* < 0.001; G4–G3: B = 2122.77, *P* = 0.010). At 1.1 mm eccentricity, the 65-79 years group had a difference in cone density with the 20–34 years and 35–49 years groups along the nasal meridians (G4–G1: B = 2611.51, *P* = 0.002; G4–G2: B = 2244.60, *P* = 0.012), with the 20–34 years and 50–64 years groups along the temporal meridians (G4–G1: B = 2420.05, *P* < 0.001; G4–G3: B = 1355.40, *P* = 0.027), and with the 35–49 years group along the superior meridian (B = 1250.64, *P* = 0.037). Along the inferior meridians, the 65-79 years group exhibited significantly lower cone density than the other 3 groups (G4–G1: B = 2762.49, *P* < 0.001; G4–G2: B = 2082.99, *P* = 0.001; G4–G3: B = 1763.16, *P* = 0.003).

### Differences in Choroidal Thickness among the 4 Groups of 4 Meridians

The choroidal thickness of each meridian in the 4 age groups is compared in Supplementary Table S2, available at www.ophthalmologyscience.org, and Figure 2B. For the nasal meridian, the mean choroidal thickness was 227.22 ± 41.56 μm (20–34 years), 198.21 ± 9.29 μm (35–49 years), 157.45 ± 15.59 μm (50–64 years), and 135.09 ± 28.21 μm (65–79 years); for the temporal meridian, it was 224.03 ± 44.46 μm (20–34 years), 199.65 ± 18.01 μm (35–49 years), 156.02 ± 16.21 μm (50–64 years), and 146.79 ± 17.81 μm (65–79 years); for the superior meridian, it was 211.16 ± 39.50 μm (20–34 years), 198.89 ± 23.26 μm (35–49 years), 155.21 ± 13.56 μm (50–64 years), and 143.91 ± 17.69 μm (65–79 years); and for the inferior meridian, it was 223.00 ± 45.11 μm (20–34 years), 198.75 ± 19.32 μm (35–49 years), 158.33 ± 14.96 μm (50–64 years), and 142.73 ± 24.93 μm (65–79 years), with the total mean choroidal thickness being 222.20 ± 40.41 μm (20–34 years), 199.45 ± 15.87 μm (35–49 years), 156.87 ± 13.45 μm (50–64 years), and 142.25 ± 20.98 μm (65–79 years). Individuals aged 35–49 years, 50–64 years, and 65–79 years showed significant choroidal thinning compared to those aged 20–34 years (G2–G1: B = 28.23, *P* = 0.003; G3–G1: B = 69.83, *P* < 0.001; G4–G1: B = 83.36, *P* < 0.001). Furthermore, the 50–64 years (G3) and 65–79 years (G4) groups presented with significantly thinner choroids than the 35–49 years group (G3–G2: B = 41.59; G4–G2: B = 55.12, all *P* < 0.001), and the nasal choroidal thickness of the 65-79 years group was additionally significantly lower than that of the 50–64 years group (G4–G3: B = 20.92, *P* = 0.045).

Meridional subgroup analysis revealed that, compared to the 20–34 years group, the 35–49 years group exhibited significant choroidal thinning, except for the superior meridian (N, B = 34.60, *P* < 0.001; T, B = 28.89, *P* = 0.005; I, B = 31.35, *P* = 0.003). The 50–64 years and 65–79 years groups demonstrated significant choroidal thinning in all 4 meridians compared to both the 20–34 years and 35–49 years groups (all *P* < 0.001; detailed B values and 95% confidence intervals are presented in Supplementary Table S4B). In addition, the 65–79 years group had a significantly thinner nasal choroid than the 50–64 years group (B = 20.92, *P* = 0.045).

### Correlation Between Cone Density, Choroidal Thickness, BCVA, and Age

**Figure 3:**
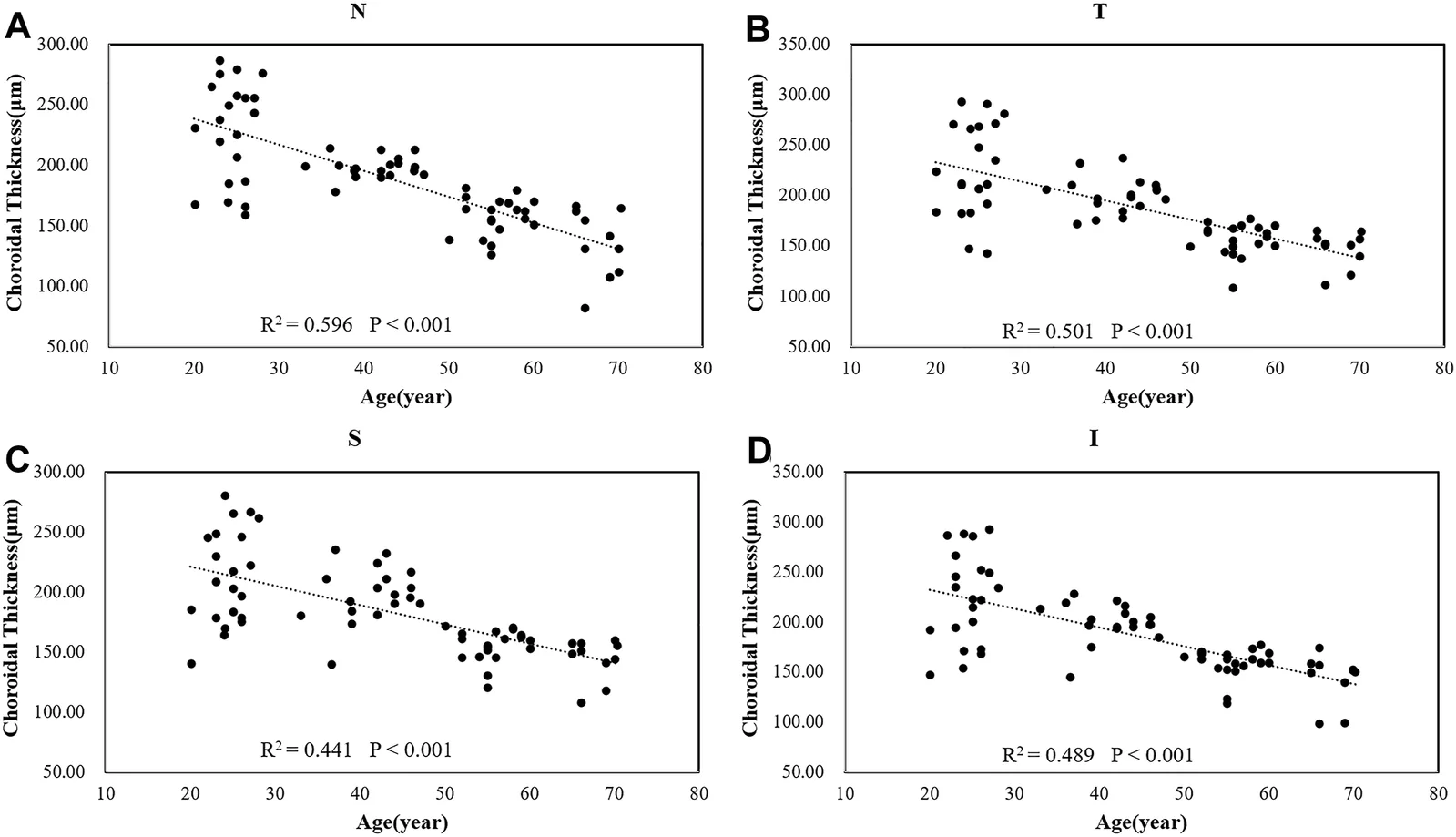
Correlation between choroidal thickness at different locations with age. **(A–D)** The choroidal thickness had significant correlations with age along the nasal **(A)**, temporal **(B)**, superior **(C)**, and inferior **(D)** meridians. I = inferior; N = nasal; S = superior; T = temporal.

**Figure 4:**
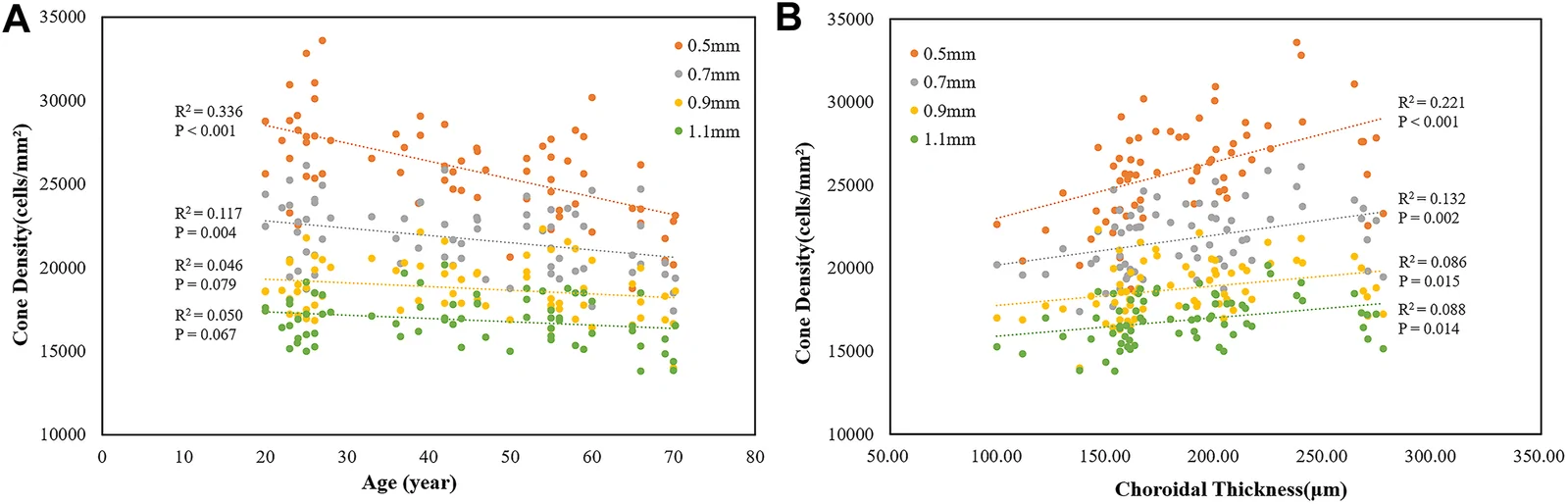
Cone density correlation with age and choroidal thickness: Pearson analysis. **(A)** The correlation between cone density and age. **(B)** The correlation between cone density and choroidal thickness. The cone density at 0.5 mm to 0.7 mm eccentricity showed correlation with age. Cone density of all eccentricities showed significant correlations with choroidal thickness.

Figure 3 illustrates the negative correlation between age and choroidal thickness along the 4 meridians (N, R^2^ = 0.596, *P* < 0.001; T: R^2^ = 0.501, *P* < 0.001; S: R^2^ = 0.441, *P* < 0.001; I: R^2^ = 0.489, *P* < 0.001). The correlation between cone density at each eccentricity, choroidal thickness, and age is depicted in Figure 4. Only the cone density at 0.5 mm to 0.7 mm eccentricity exhibited a correlation with age (Fig 4A, 0.5 mm: R^2^ = 0.336, *P* < 0.001; 0.7 mm: R^2^ = 0.117, *P* = 0.004). The correlation between cone density and age for each eccentricity along the 4 meridians is displayed in Supplementary Table S3 (available at www.ophthalmologyscience.org). A significantly negative relationship was observed between age and cone density from 0.5 mm to 1.1 mm eccentricity at the nasal meridian (0.5 mm: R^2^ = 0.311, *P* < 0.001; 0.7 mm: R^2^ = 0.200, *P* < 0.001; 0.9 mm: R^2^ = 0.077, *P* = 0.024; 1.1 mm: R^2^ = 0.077, *P* = 0.024). At the temporal, superior, and inferior meridians, cone density at eccentricities of 0.5 mm from the fovea was significantly correlated with age (temporal: R^2^ = 0.324, *P* < 0.001; superior: R^2^ = 0.141, *P* = 0.002; inferior: R^2^ = 0.186, *P* < 0.001). At 0.7 mm eccentricity, only the temporal meridian exhibited a correlation with age (R^2^ = 0.073, *P* = 0.027). The overall averaged cone density (whole) was significantly correlated with age at 0.5 mm and 0.7 mm eccentricities (0.5 mm: R^2^ = 0.336, *P* < 0.001; 0.7 mm: R^2^ = 0.117, *P* = 0.004). In summary, cone density was negatively related to age at 0.5 mm eccentricities across all meridians, with the nasal meridian showing the most extended age-related decline up to 1.1 mm.

Cone density at all eccentricities demonstrated a significantly positive relationship with choroidal thickness (Fig 4B). Compared to 0.7 mm to 1.1 mm eccentricity, cone density at 0.5 mm eccentricity exhibited stronger correlations with choroidal thickness (0.5 mm: R^2^ = 0.221, *P* < 0.001; 0.7 mm: R^2^ = 0.132, *P*=0.002; 0.9 mm: R^2^ = 0.086, *P* = 0.015; 1.1 mm: R^2^ = 0.088, *P* = 0.014). Furthermore, significant correlations were discovered between cone density and BCVA (Table 2; 0.5 mm: R^2^ = 0.372, *P* < 0.001; 0.7 mm: R^2^ = 0.259, *P* < 0.001; 0.9 mm: R^2^ = 0.255, *P* < 0.001; 1.1 mm: R^2^ = 0.211, *P* < 0.001).

**Table 2:** The Correlation of Cone Density at Different Eccentricities with BCVA

Eccentricity	R^2^	*P* Value
0.5 mm	0.372	**<0.001**
0.7 mm	0.259	**<0.001**
0.9 mm	0.255	**<0.001**
1.1 mm	0.211	**<0.001**

The bold values represent *P* values <0.05.

BCVA = best-corrected visual acuity.

## Discussion

This study employed an AO fundus camera and spectral domain OCT to quantitatively assess age-related morphological changes in the photoreceptor layer (cone density) and choroidal vasculature (choroidal thickness). The aim was to investigate how choroidal blood supply and retinal structure covary with advancing age, and to further explore their functional correlate—visual acuity. By integrating these 3 dimensions, we aimed to elucidate the interconnections among vascular support, tissue structure, and visual function over the lifespan. A deeper understanding of the interconnections among them might provide critical insights into the mechanisms of visual impairment and serve as a foundational reference for studying age-related chorioretinopathy.

The current study discovered that the foveal choroidal thickness decreases by 1.91 μm per year. After correcting for AL and sex, the results suggested that choroidal thickness in the eyes of individuals over 51 years old was significantly lower than that of the age group younger than 50 years old. The thickness, after 60 years of age, underwent further thinning at the nasal meridian. These results are consistent with those of previous studies (Table 3). Margolis R et al^10^ found that the subfoveal choroid decreased 1.56 μm for each year of age. Fujiwara et al^12^ showed that subfoveal choroidal thickness in Japanese participants decreases by 2.0 μm each year after 30 years old.

**Table 3:** Summary of Previous Studies on Choroid and Cone with Age

Parameter	Study	Age, yrs.	SE, D	Instruments	Alteration Related to Age
	Current study	20 ∼ 70	–3.25 ∼ +0.63	AO fundus camera, rtx1 SD-OCT, Optovue RTVue	The foveal choroidal thickness decreases by 1.91 μm per year. The cone density reduced with age, which was decreased from approximately 27.9 × 10^3^ cones/mm^2^ (aged 20–34 years) to 22.3 × 10^3^ cones/mm^2^ (aged over 65 years) at 0.5 mm eccentricity, which was similar at other degrees of eccentricity.
Choroid thickness	Margolis R et al^10^	19 ∼ 85	–1.3 ± 2.1 (mean SE)	EDI OCT	The choroidal thickness decreased by 1.56 μm per year of age.
	Fujiwara T et al^12^	24 ∼ 90	–11.9 ± 3.7 (mean SE)	EDI OCT	The subfoveal choroidal thickness decreased by 1.27 μm per year of age.
Cone density	Song H et al^20^	22 ∼ 65	–2.0 ∼ +3.5	AOSLO	The cone density reduced with age. The cone density of younger group was from 16.1 × 103 to 25.6 × 103 cones/mm2 and the older group was from 15.9 × 103 to 22.4 × 103 cones/mm2 at the eccentricity of 0.81 mm.
	Legras R et al^15^	20 ∼ 59	–5.50 ∼ +3.50	AO fundus camera, rtx1	The cone density was around 31 000 cones/mm^2^ with the age from 18 to 30 years, and reduced with age to approximately 27 000 cones/mm^2^ at 2° of eccentricity. This tendency can be observed at other degrees of eccentricity.
	Park et al^3^	10 ∼ 69	–9.8 ∼ +2.5	AOSLO	The cone density decreased from 32 199 cones/mm^2^ (0.5 mm eccentricity) to 11 597 cones/mm^2^ (1.5 mm eccentricity) and had no significant correlation with age.

AO = adaptive optics; AOSLO = adaptive optics scanning laser ophthalmoscopy; EDI-OCT = enhanced depth imaging spectral-domain OCT; SD = spectral-domain OCT; SE = spherical equivalent.

Aging has an effect on cone alterations, some directly affecting the cone density while others are possible underlying mechanisms of cone decline.^3^^,^^15^^,^^20^^,^^21^ Song et al^20^ found that cone density was significantly lower in an older group than in a younger group and was related to retinal eccentricity. Gartner et al^21^ found a noticeable reduction in the number of cone nuclei in 24 of 104 necropsy eyes of patients over 40 years of age. Conversely, Sung et al^3^ found no statistical correlation between cone density and age. The cone decline is thought to be primarily due to decreased cone photopigment at the center of the fovea^22^ and age-related photoreceptor nuclei loss.^21^

In the present study, the cone density of the entire age group was reduced in the nasal meridian at all eccentricities, indicating that the nasal meridian was more susceptible; however, the specific mechanism needs further investigation. Additionally, the cone density at 0.5 mm eccentricities from the fovea along the 4 meridians all showed a significant correlation with age. Moreover, our results support the notion that cone density reduction has regional characteristics across different age groups. Individuals aged 50–64 years had lower cone density at foveal 0.5 mm to 0.7 mm eccentricity, and those aged 65–79 years had significantly lower cone density within 0.5 mm eccentricity from the fovea than people under 35. We speculate that age mainly affects the cone density within 0.7 mm eccentricity from the age of 50 years, and the influence range narrows when the age exceeds 60 years. This study analyzed and compared the cone density of different age groups and expressed the cone density in more detail according to the fundus quadrants and macular eccentricity.

Damage to the cone photoreceptor is crucial in the pathophysiology of various chorioretinopathies,^5^, ^6^, ^7^^,^^13^ resulting in a decline in visual function. Disorders of the photoreceptors have been shown to correlate with a decrease in macular light sensitivity, which is also associated with chorioretinal degeneration.^14^ Due to its highly vascular structure, the choroid is considered the most important blood supply organ and is crucial for visual function.^8^ The present study discovered that the decreased cone density in participants over 50 years of age may be related to photoreceptor degeneration associated with age-related choroidal thinning.”^21^ One possible explanation is that age-related reduction in peripapillary choroidal thickness may contribute to insufficient blood supply to the retinal pigment epithelium, outer retina, and even choroidal stroma, which could potentially be associated with peripapillary choroidal atrophy, retinal pigment epithelium atrophy, and photoreceptor degeneration.^8^, ^9^, ^10^, ^11^, ^12^ Similarly, Wei et al^23^ indicated a connection between age and retinal microstructure, microvasculature, and microcirculation, and hypothesized that thinning of the retinal layer occurred with the loss of retinal microvessel density during normal aging.

In addition, previous research showed that the reduction in choroidal perfusion is correlated with irreversible and progressive damage to physiological functions, including visual acuity.^8^^,^^24^ The current study speculated that the age-related gradual thinning of the choroid led to reduced blood perfusion and oxygen supply to the retina^8^^,^^25^, resulting in the continuous damage of cone photoreceptors that eventually leads to vision loss. A comprehensive understanding of the changing characteristics of normal aging is essential for disease evaluation. The results offer insightful information for understanding the occurrence of chorioretinopathy, photoreceptor damage, and the mechanisms of visual impairment.

This study possesses certain limitations. First, although we acquired a relatively large sample with a wide range of ages, the sample size was still insufficient, and participants older than 70 years may also need to be included. Second, we did not conduct longitudinal research, which could have revealed the order and temporal trends of these changes during aging. Further longitudinal studies are required to confirm our findings. Third, choroidal thickness was automatically measured with a custom algorithm and adjusted manually when segmentation errors occurred. Potential bias introduced by manual correction should be considered. Besides, although the cone counting method we used relies on a highly automated algorithm, which minimizes operator-induced variability, and prior studies have established the high reproducibility of this system, we did not conduct a formal intergrader reliability test for this specific study cohort. The lack of a data set–specific reliability assessment represents a limitation. Fourth, compared to the adaptive optics scanning laser ophthalmoscopy, the axial resolution and contrast of the images captured by AO fundus camera (rtx1) are worse than those of adaptive optics scanning laser ophthalmoscopy, especially the resolution within 0.5 mm eccentricity of the fovea. Otherwise, the minor axial alignment deviations in AO imaging may cause local image blur, which could indirectly cause the underestimation of the cone density. These are considered to be the reason for the low cone density in current study. Besides, because of the limited adaptive optical processing of the AO camera, we only investigated a 3 mm × 3 mm area. Further investigation needs to expand the measurement range and enhance the measurement accuracy. Finally, BCVA may not indicate early changes in visual function during aging. Other visual functional measurements are essential for evaluating the microstructural and functional consequences.

## Conclusions

In conclusion, the application of AO fundus cameras and OCT enabled a multidimensional assessment of choroidal vascular supply, photoreceptor structure, and their functional output during aging. The results demonstrated that the cone density is significantly associated with both age and the choroidal thickness, suggesting that age serves as a common driver linking blood supply, structural integrity, and functional decline. An accurate understanding of the changing characteristics of the human eye during aging is a prerequisite for exploring the progression of age-related ocular diseases. Elucidating how vascular, structural, and functional parameters evolve together may facilitate early clinical identification, support timely intervention in age-related chorioretinopathy, and enhance the evaluation of visual impairment in the aging population.
