# Supplementary material for: Associations of Cone Density and Choroidal Thickness with Age: A Cross-Sectional Study
Source: Ophthalmol Sci. 2026 Jul 23;6(10):101329. doi: 10.1016/j.xops.2026.101329 (PMC13545361; doi:10.1016/j.xops.2026.101329)
Supplement: Supplementary file 1 [file mmc1.pdf]

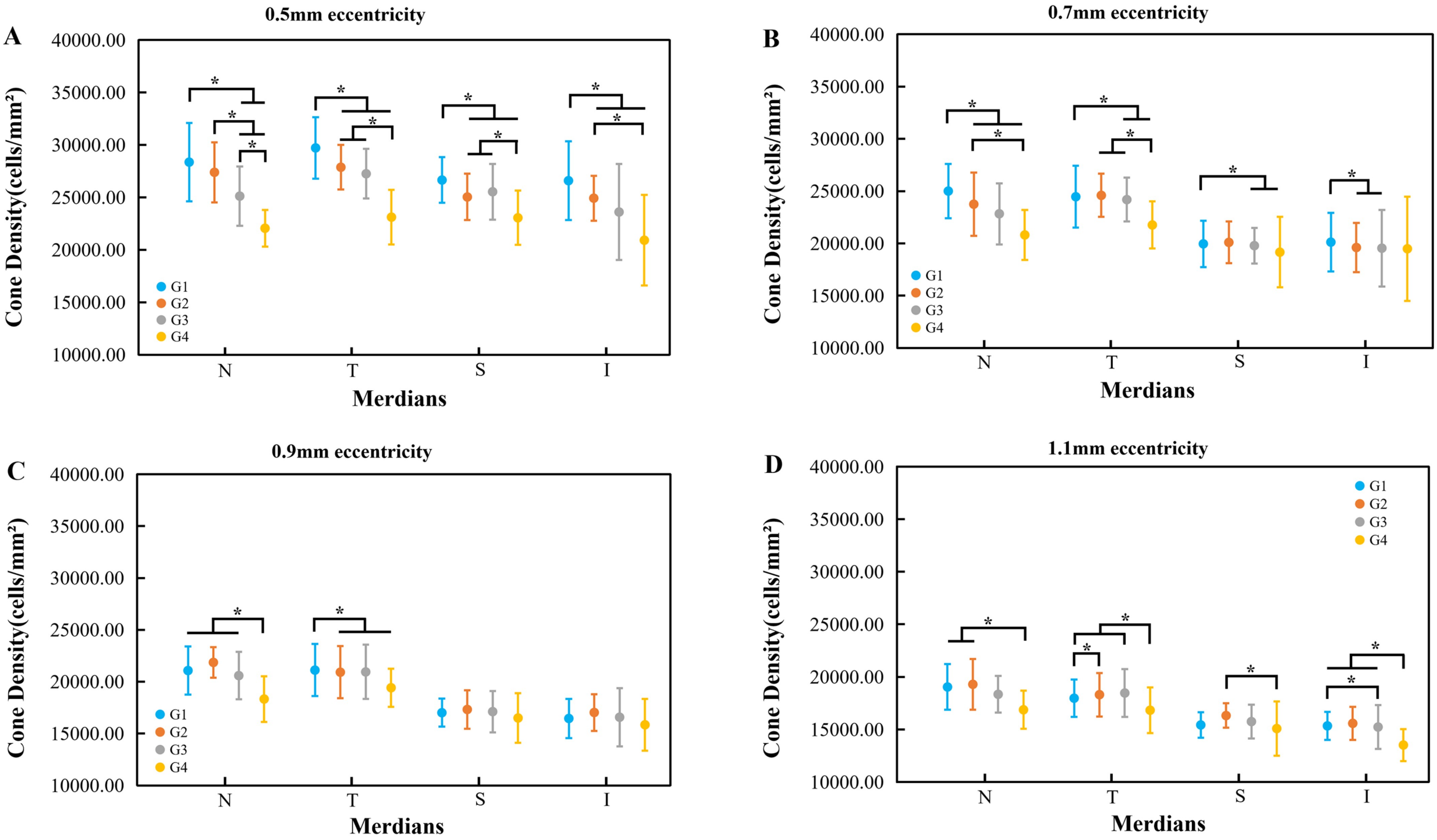
A
0.5mm eccentricity
B
0.7mm eccentricity
C
0.9mm eccentricity
D
1.1mm eccentricity
Cone Density(cells/mm²)
40000.00
35000.00
30000.00
25000.00
20000.00
15000.00
10000.00
N
T
S
I
Merdians
G1
G2
G3
G4
*
