# Supplementary material for: Associations of Cone Density and Choroidal Thickness with Age: A Cross-Sectional Study
Source: Ophthalmol Sci. 2026 Jul 23;6(10):101329. doi: 10.1016/j.xops.2026.101329 (PMC13545361; doi:10.1016/j.xops.2026.101329)
Supplement: Supplementary file 2 [file mmc2.pdf]

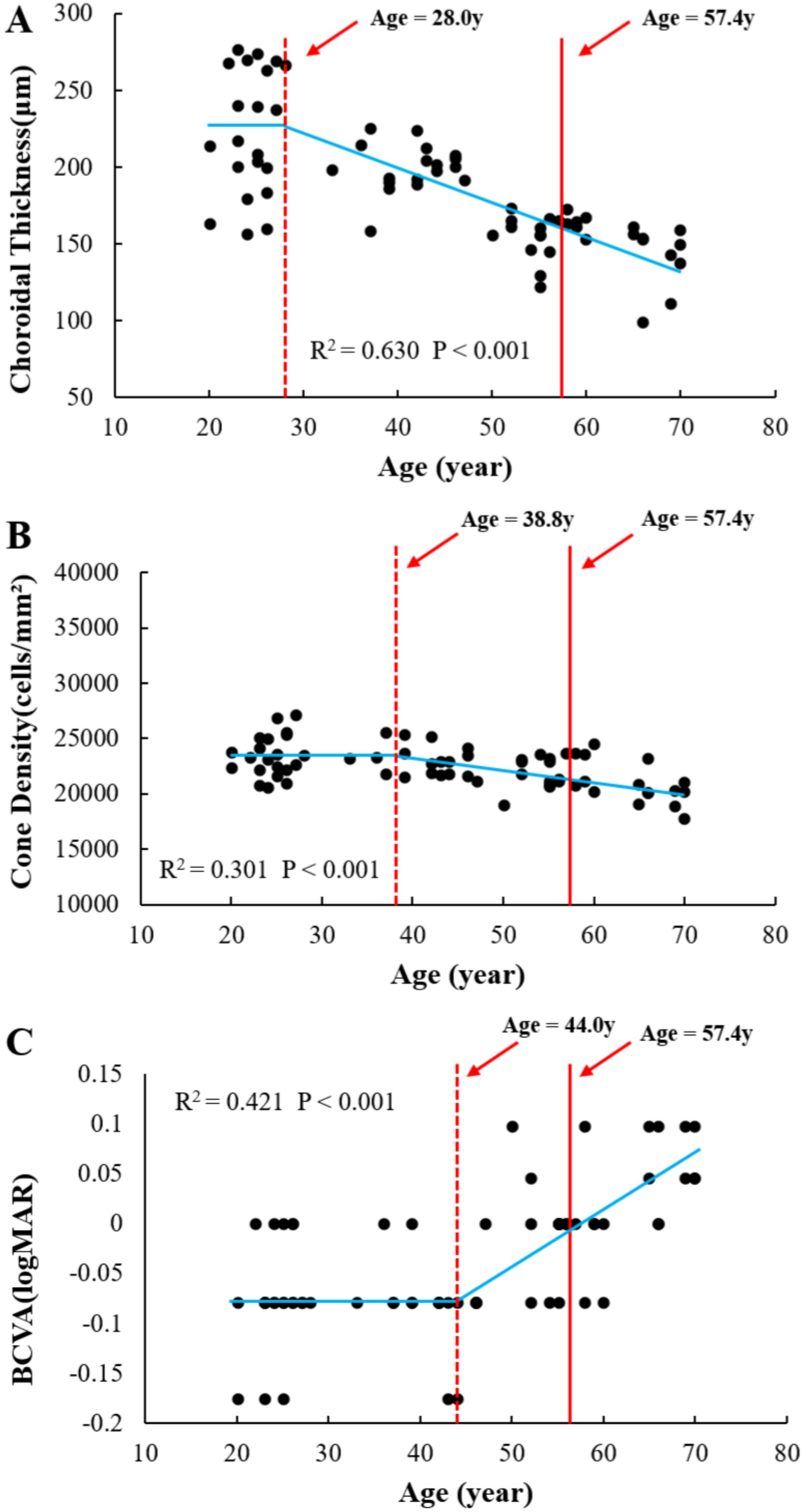
A
Age = 28.0y
Age = 57.4y
Choroidal Thickness(μm)
R2 = 0.630 P < 0.001
Age (year)
B
Age = 38.8y
Age = 57.4y
Cone Density(cells/mm2)
R2 = 0.301 P < 0.001
Age (year)
C
Age = 44.0y
Age = 57.4y
BCVA(logMAR)
R2 = 0.421 P < 0.001
Age (year)
