# Supplementary material for: Associations of Cone Density and Choroidal Thickness with Age: A Cross-Sectional Study
Source: Ophthalmol Sci. 2026 Jul 23;6(10):101329. doi: 10.1016/j.xops.2026.101329 (PMC13545361; doi:10.1016/j.xops.2026.101329)
Supplement: Supplementary file 3 [file mmc3.pdf]

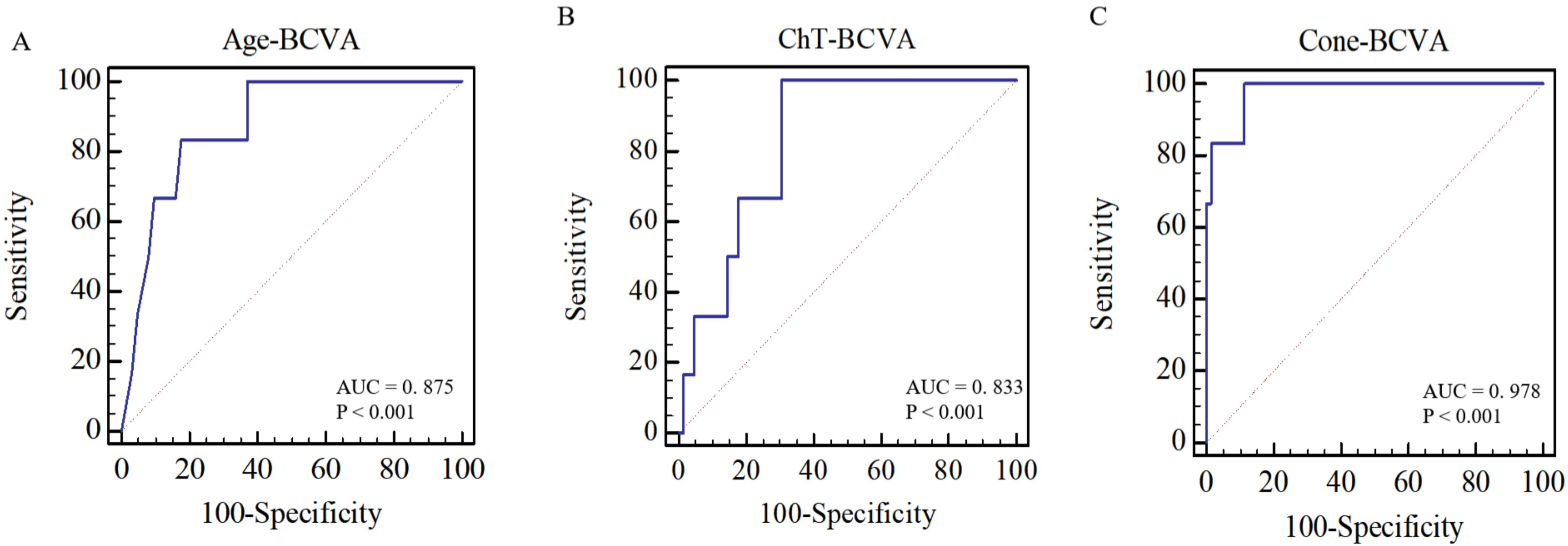

A
Age-BCVA
AUC = 0. 875
P < 0.001
B
ChT-BCVA
AUC = 0. 833
P < 0.001
C
Cone-BCVA
AUC = 0. 978
P < 0.001
Sensitivity
100-Specificity
0
20
40
60
80
100
