# Supplementary material for: Associations of Cone Density and Choroidal Thickness with Age: A Cross-Sectional Study
Source: Ophthalmol Sci. 2026 Jul 23;6(10):101329. doi: 10.1016/j.xops.2026.101329 (PMC13545361; doi:10.1016/j.xops.2026.101329)
Supplement: Supplementary file 4 [file mmc4.pdf]

Supplementary Table S1. Mean cone density at different locations in four groups of different age

		G1	G2	G3	G4
Nasal	0.5mm	28.36 ± 3.74	27.39 ± 2.86	25.12 ± 2.84*†	22.06 ± 1.75*†‡
	0.7mm	25.00 ± 2.61	23.75 ± 3.03*	22.83 ± 2.92*	20.81 ± 2.40*†
	0.9mm	21.08 ± 2.31	21.86 ± 1.48	20.59 ± 2.30	18.33 ± 2.20*†‡
	1.1mm	19.04 ± 2.18	19.29 ± 2.41	18.35 ± 1.75	16.87 ± 1.82*†
Temporal	0.5mm	29.72 ± 2.94	27.88 ± 2.12*	27.26 ± 2.36*	23.12 ± 2.61*†‡
	0.7mm	24.46 ± 2.96	24.60 ± 2.07	24.19 ± 2.09*	21.76 ± 2.25*†‡
	0.9mm	21.12 ± 2.52	20.91 ± 2.50*	20.94 ± 2.62*	19.42 ± 1.84*
	1.1mm	17.97 ± 1.79	18.31 ± 2.07*	18.47 ± 2.27	16.83 ± 2.18*‡
Superior	0.5mm	26.65 ± 2.17	25.04 ± 2.21*	25.54 ± 2.65*	23.06 ± 2.60*†‡
	0.7mm	19.96 ± 2.22	20.10 ± 1.98	19.78 ± 1.70*	19.16 ± 3.37*
	0.9mm	17.01 ± 1.35	17.32 ± 1.85	17.11 ± 1.99	16.50 ± 2.39
	1.1mm	15.43 ± 1.21	16.32 ± 1.15	15.75 ± 1.61	15.09 ± 2.58†
Inferior	0.5mm	26.60 ± 3.74	24.93 ± 2.14*	23.61 ± 4.58*	20.92 ± 4.32*†
	0.7mm	20.12 ± 2.81	19.61 ± 2.35*	19.54 ± 3.66*	19.49 ± 4.98
	0.9mm	16.46 ± 1.89	17.03 ± 1.77	16.58 ± 2.80	15.85 ± 2.50
	1.1mm	15.34 ± 1.35	15.58 ± 1.56	15.23 ± 2.07*	13.52 ± 1.52*†‡
Whole	0.5mm	27.86±2.77	26.31±1.54*	25.33±2.44*†	22.29±2.10*†‡
	0.7mm	22.43±2.04	22.01±1.66*	21.52±2.02*	20.30±2.05*†
	0.9mm	18.91±1.42	19.28±1.37	18.86±1.89*	17.50±1.67*†‡
	1.1mm	16.95±1.19	17.40±1.38	17.05±1.29*	15.56±1.45*†‡

Densities are expressed as ×1000 cells/mm² (mean ± SD).

*Significant compared to Group1. †Significant compared to Group2. ‡Significant compared to Group3.

Analysis of the generalized linear model, $P<0.05$.
