# Supplementary material for: Associations of Cone Density and Choroidal Thickness with Age: A Cross-Sectional Study
Source: Ophthalmol Sci. 2026 Jul 23;6(10):101329. doi: 10.1016/j.xops.2026.101329 (PMC13545361; doi:10.1016/j.xops.2026.101329)
Supplement: Supplementary file 5 [file mmc5.pdf]

Supplementary Table S2. Mean Choroidal Thickness at Different Locations in Four Groups of Different Age

	G1	G2	G3	G4
Nasal	227.22 ± 41.56	198.21 ± 9.29*	157.45 ± 15.59*†	135.09 ± 28.21*†‡
Temporal	224.03 ± 44.46	199.65 ± 18.01*	156.02 ± 16.21*†	146.79 ± 17.81*†
Superior	211.16 ± 39.50	198.89 ± 23.26	155.21 ± 13.56*†	143.91 ± 17.69*†
Inferior	223.00 ± 45.11	198.75 ± 19.32*	158.33 ± 14.96*†	142.73 ± 24.93*†
Total	222.20 ± 40.41	199.45 ± 15.87*	156.87 ± 13.45*†	142.25 ± 20.98*†

*Significant compared to Group1. †Significant compared to Group2. ‡Significant compared to Group3.

An analysis of generalized linear model, $P<0.05$.
