# Supplementary material for: Associations of Cone Density and Choroidal Thickness with Age: A Cross-Sectional Study
Source: Ophthalmol Sci. 2026 Jul 23;6(10):101329. doi: 10.1016/j.xops.2026.101329 (PMC13545361; doi:10.1016/j.xops.2026.101329)
Supplement: Supplementary file 6 [file mmc6.pdf]

Supplementary Table S3. Pearson Correlation Analysis Age Associated with Cone Density in All Participants (N=68)

Eccentricity		0.5mm	0.7mm	0.9mm	1.1mm
Nasal	R^2	0.311	0.200	0.077	0.077
	P value	**<0.001**	**<0.001**	**0.024**	**0.024**
Temporal	R^2	0.324	0.073	0.031	0.007
	P value	**<0.001**	**0.027**	0.149	0.508
Superior	R^2	0.141	0.015	0.007	0.001
	P value	**0.002**	0.318	0.501	0.821
Inferior	R^2	0.186	0.010	0.002	0.062
	P value	**<0.001**	0.418	0.737	0.053
whole	R^2	0.336	0.117	0.046	0.050
	P value	**<0.001**	**0.004**	0.079	0.067

The bold values represented *P* value < 0.05
