# Supplementary material for: Associations of Cone Density and Choroidal Thickness with Age: A Cross-Sectional Study
Source: Ophthalmol Sci. 2026 Jul 23;6(10):101329. doi: 10.1016/j.xops.2026.101329 (PMC13545361; doi:10.1016/j.xops.2026.101329)
Supplement: Supplementary file 7 [file mmc7.pdf]

Supplementary Table S4A. Generalized linear model analysis of cone density at different eccentricities and regions among different age groups

Location	Eccentricity (mm)	Omnibus test statistic (LR χ^2)	df	P (overall)	G2–G1: B (95% CI); P_adj	G3–G1: B (95% CI); P_adj	G4–G1: B (95% CI); P_adj	G3–G2: B (95% CI); P_adj	G4–G2: B (95% CI); P_adj	G4–G3: B (95% CI); P_adj
Nasal	0.5	38.49	5	**<0.001**	1906.78 (-77.25~3890.80); 0.060	4639.22 (2710.79~6567.66); **<0.001**	7569.32 (5362.37~9776.27); **<0.001**	2732.45 (789.37~4675.52); **0.006**	5662.54 (3347.97~7977.11); **<0.001**	2930.10 (780.91~5079.29); **0.008**
	0.7	20.42	5	**0.001**	2180.38 (304.76~4056.00); **0.023**	2949.04 (1152.65~4745.44); **0.001**	4794.83 (2706.86~6882.80); **<0.001**	768.67 (-1053.66~2590.99); 0.408	2614.45 (425.17~4803.74); **0.019**	1845.79 (-166.02~3857.59); 0.072
	0.9	20.99	5	**0.001**	32.17 (-1371.92~1436.25); 0.964	1174.62 (-169.96~2519.20); 0.087	3297.39 (1621.75~4973.04); **<0.001**	1142.45 (-221.66~2506.56); 0.101	3265.22 (1527.02~5003.43); **<0.001**	2122.77 (501.79~3743.76); **0.010**
	1.1	12.17	5	**0.032**	366.92 (-1046.90~1780.73); 0.611	1242.67 (-111.23~2596.57); 0.072	2611.51 (924.26~4298.77); **0.002**	875.76 (-497.81~2249.32); 0.211	2244.60 (494.35~3994.84); **0.012**	1368.84 (-263.38~3001.06); 0.100
Temporal	0.5	46.78	5	**<0.001**	2676.53 (1023.77~4329.28); **0.002**	3614.07 (2031.12~5197.01); **<0.001**	7643.22 (5803.34~9483.09); **<0.001**	937.54 (-668.26~2543.33); 0.252	4966.69 (3037.54~6895.84); **<0.001**	4029.15 (2256.39~5801.91); **<0.001**
	0.7	25.53	5	**<0.001**	1296.25 (-214.11~2806.61); 0.093	1549.77 (65.67~3033.87); **0.041**	3676.39 (3033.87~5361.88); **<0.001**	253.52 (-1255.76~1762.80); 0.742	2380.13 (611.70~4148.57); **0.008**	2126.62 (492.93~3760.31); **0.011**
	0.9	30.24	5	**<0.001**	1935.30 (525.74~3344.87); **0.007**	1858.92 (508.89~3208.95); **0.007**	3069.40 (1500.24~4638.55); **<0.001**	-76.38 (-1445.90~1293.13); 0.913	1134.09 (-511.20~2779.39); 0.177	1210.48 (-301.44~2722.39); 0.117

	1.1	38.17	5	**<0.001**	1233.94<br>(113.43~2354.46);<br>**0.031**	1064.64<br>(-8.54~2137.83);<br>0.052	2420.05<br>(1172.67~3667.43);<br>**<0.001**	-169.30<br>(-1257.98~919.38);<br>0.761	1186.10<br>(-121.80~2494.01);<br>0.075	1355.40<br>(153.52~2557.28);<br>**0.027**
Superior	0.5	44.31	5	**<0.001**	2558.59<br>(1223.40~3893.79);<br>**<0.001**	2802.84<br>(1506.95~4098.74);<br>**<0.001**	5204.19<br>(3718.54~6689.83);<br>**<0.001**	244.25<br>(-1063.70~1552.21);<br>0.714	2645.60<br>(1087.45~4203.74);<br>**0.001**	2401.34<br>(954.85~3847.84);<br>**0.001**
	0.7	18.60	5	**0.002**	1033.71<br>(-338.35~2405.78);<br>0.140	1502.36<br>(188.25~2816.7);<br>**0.025**	1939.23<br>(411.82~3466.63);<br>**0.013**	468.65<br>(-864.43~1801.72);<br>0.491	905.51<br>(-696.00~2507.03);<br>0.268	436.87<br>(-1034.82~1908.56);<br>0.561
	0.9	12.92	5	**0.024**	391.66<br>(-785.35~1568.68);<br>0.514	778.26<br>(-349.95~1906.48);<br>0.176	1278.17<br>(-34.17~2590.51);<br>0.056	386.60<br>(-757.24~1530.43);<br>0.508	886.51<br>(-488.40~2261.42);<br>0.206	499.91<br>(-762.72~1762.54);<br>0.438
	1.1	16.11	5	**0.007**	-238.07<br>(-1247.10~770.96);<br>0.644	449.88<br>(-516.53~1416.30);<br>0.362	1012.57<br>(-110.71~2135.84);<br>0.077	687.95<br>(-292.41~1668.32);<br>0.169	1250.64<br>(72.86~2428.41);<br>**0.037**	562.68<br>(-519.62~1644.98);<br>0.308
Inferior	0.5	27.18	5	**<0.001**	3536.93<br>(1066.66~6007.20);<br>**0.005**	4877.22<br>(2518.27~7236.16);<br>**<0.001**	7281.41<br>(4583.45~9979.37);<br>**<0.001**	1340.29<br>(-1017.30~3697.88);<br>0.265	3744.48<br>(932.62~6556.34);<br>**0.009**	2404.19<br>(-197.46~5005.85);<br>0.070
	0.7	14.03	5	**0.015**	2502.05<br>(380.11~4623.99);<br>**0.021**	2283.89<br>(275.35~4292.43);<br>**0.026**	2073.46<br>(-416.77~4563.69);<br>0.103	-218.16<br>(-2225.60~1789.29);<br>0.831	-428.58<br>(-2986.35~2129.18);<br>0.743	-210.43<br>(-2589.88~2169.03);<br>0.862
	0.9	11.52	5	**0.042**	526.57<br>(-910.66~1963.80);<br>0.473	729.27<br>(-650.24~2108.78);<br>0.300	1342.30<br>(-379.10~3063.69);<br>0.126	202.70<br>(-1203.35~1608.74);<br>0.778	815.73<br>(-961.00~2592.45);<br>0.368	613.03<br>(-1056.46~2282.52);<br>0.472
	1.1	26.57	5	**<0.001**	679.50	999.33	2762.49	319.83	2082.99	1763.16

					(-316.68~1675.67); 0.181	(20.66~1978.00); **0.045**	(1573.79~3951.18); **<0.001**	(-680.05~1319.70); 0.531	(863.17~3302.81); **0.001**	(586.44~2939.88); **0.003**
	0.5	55.12	5	**<0.001**	2685.92 (1301.33~4070.50); **<0.001**	4038.41 (2712.31~5364.52); **<0.001**	6929.87 (5388.53~8471.22); **<0.001**	1352.50 (7.25~2697.74); **0.049**	4243.96 (2627.82~5860.09); **<0.001**	2891.46 (1406.34~4376.58); **<0.001**
Whole	0.7	33.59	5	**<0.001**	1788.23 (657.07~2919.40); **0.002**	2196.06 (1112.68~3279.44); **<0.001**	3161.00 (1901.77~4420.23); **<0.001**	407.82 (-691.19~1506.85); 0.467	1372.77 (52.44~2693.10); **0.042**	964.94 (-248.35~2178.24); 0.119
	0.9	33.63	5	**<0.001**	734.33 (-184.16~1652.81); 0.117	1109.85 (230.16~1989.54); **0.013**	2277.99 (1255.52~3300.47); **<0.001**	375.52 (-516.87~1267.91); 0.410	1543.67 (471.58~2615.75); **0.005**	1168.14 (182.96~2153.32); **0.02**
	1.1	42.78	5	**<0.001**	476.25 (-252.40~1204.91); 0.200	858.09 (160.21~1555.98); **0.016**	2182.37 (1371.21~2993.53); **<0.001**	381.84 (-326.12~1089.79); 0.290	1706.12 (855.60~2556.63); **<0.001**	1324.28 (542.71~2105.84); **0.001**

Analysis of the generalized linear model, the bold values represented P value < 0.05.

Supplementary Table S4B. Generalized linear model analysis of choroidal thickness at different regions among different age groups

Region	Omnibus test statistic	df	P (overall)	G2–G1: B (95% CI); P_adj	G3–G1: B (95% CI); P_adj	G4–G1: B (95% CI); P_adj	G3–G2: B (95% CI); P_adj	G4–G2: B (95% CI); P_adj	G4–G3: B (95% CI); P_adj
Nasal	68.20	5	**<0.001**	34.60 (15.57~53.64); **<0.001**	71.66 (53.43~89.89); **<0.001**	92.58 (71.39~113.77); **<0.001**	37.06 (18.56~55.55); **<0.001**	57.98 (35.76~80.20); **<0.001**	20.92 (0.50~41.34); **0.045**
Temporal	55.76	5	**<0.001**	28.89 (8.75~49.02); **0.005**	71.89 (52.61~91.17); **<0.001**	80.25 (57.84~102.67); **<0.001**	43.00 (23.44~62.56); **<0.001**	51.37 (27.87~74.87); **<0.001**	8.36 (-13.23~29.96); 0.448
Superior	51.46	5	**<0.001**	16.60 (-2.18~35.37); 0.083	60.87 (42.89~78.85); **<0.001**	71.48 (50.58~92.38); **<0.001**	44.27 (26.03~62.51); **<0.001**	54.88 (32.96~76.79); **<0.001**	10.61 (-9.53~30.74); 0.302
Inferior	54.13	5	**<0.001**	31.35 (10.74~51.96); **0.003**	71.86 (52.12~91.60); **<0.001**	86.21 (63.26~109.16); **<0.001**	40.51 (20.48~60.54); **<0.001**	54.86 (30.79~78.92); **<0.001**	14.35 (-7.76~36.46); 0.203
Total	63.24	5	**<0.001**	28.23 (9.89~46.58); **0.003**	69.83 (52.26~87.40); **<0.001**	83.36 (62.93~103.78); **<0.001**	41.59 (23.77~59.42); **<0.001**	55.12 (33.71~76.54); **<0.001**	13.53 (-6.15~33.21); 0.178

Analysis of the generalized linear model, the bold values represented P value < 0.05.
